# Exploring the predictors affecting the sense of community of Korean high school students: application of random forests and SHAP

**DOI:** 10.3389/fpsyg.2024.1337512

**Published:** 2024-02-06

**Authors:** Eunah Jang, Hyewon Chung

**Affiliations:** Department of Education, Chungnam National University, Daejeon, Republic of Korea

**Keywords:** sense of community, machine learning, random forests, SHapley Additive exPlanations, Korean high school students

## Abstract

Adolescence is a stage during which individuals develop social adaptability through meaningful interactions with others. During this period, students gradually expand their social networks outside the home, forming a sense of community. The aim of the current study was to explore the key predictors related to sense of community among Korean high school students and to develop supportive policies that enhance their sense of community. Accordingly, random forests and SHapley Additive exPlanations (SHAP) were applied to the 7th wave (11th graders) of the Korean Education Longitudinal Study 2013 data (*n* = 6,077). As a result, 6 predictors positively associated with sense of community were identified, including self-related variables, “multicultural acceptance,” “behavioral regulation strategy,” and “peer attachment,” consistent with previous findings. Newly derived variables that predict sense of community include “positive recognition of volunteering,” “creativity,” “observance of rules” and “class attitude,” which are also positively related to sense of community. The implications of these results and some suggestions for future research are also discussed.

## 1 Introduction

Adolescence is the stage of developing self-identity when individuals gradually begin to recognize themselves as independent beings and develop social adaptability through meaningful interactions with others (Erikson, 1968). Social development or adaptability, the ability to build and maintain interpersonal relationships according to the norms of an individual’s community, is one of the developmental tasks of adolescence and an essential part of becoming a healthy and mature member of society (Ranju and Manisha, 2015). During this period, students gradually expand their social networks outside the home, forming a sense of community. Thus, the adaptive aspect of adolescent social development manifests through a sense of community. In other words, adolescents develop a variety of relationships with their teachers and peers in school life and form perceptions of their school community, regional community, and nation, which in turn leads to their sense of community. A sense of community reflects the ability to recognize a network of mutually supportive relationships among the members of a community; thus, the development of a sense of community in adolescence is particularly important because it leads to citizenship and social engagement in adulthood (Sarason, 1974).

However, there are growing concerns regarding Korean adolescents; living in hypercompetitive societies, they currently have a strong sense of selfishness and individualism as well as a tendency toward isolation and disconnection from each other. Additionally, they show a noticeable lack of communal ethics, such as care, attachment and responsibility for others (Lee, 2006). In particular, Korean high school students are exposed to a more competitive entrance examination atmosphere than middle school students are, which can lead to excessive competition and heavy learning demands, making it difficult for them to develop social skills (Yoon and Sung, 2021). On the other hand, given that empirical studies have shown that a sense of community may promote high school students’ well-being, such as their satisfaction with life (Hombrados-Mendieta et al., 2019), mental health (Terry et al., 2019) and happiness (Lee and Cho, 2023), it is necessary to identify the factors that have meaningful impacts on fostering a sense of community among high school students and developing supportive policies that enhance this sense of community.

### 1.1 The current study

Studies on the variables that affect sense of community have reported various student-related variables, such as “gender,” “self-concept,” “self-esteem,” “career maturity,” “life satisfaction,” “academic achievement” and “internet addiction” (Cantarero et al., 2007; Kissinger et al., 2009; Wighting et al., 2015; Min, 2017; Jung et al., 2018); family-related variables, such as “household income,” “parental education,” “parental social support” and “parental rearing behavior” (Vieno et al., 2007; Zaff et al., 2008; Park, 2019); and school-related variables, such as “social support from peers,” “social support from teachers,” “school culture,” and “democratic school climate” (Vieno et al., 2005; Kang and Jang, 2013; Kim and Kim, 2015), which are associated with adolescents’ sense of community.

However, the above studies are similarly limited; they have all employed classical statistical modeling, such as correlation analysis, structural equation modeling and multilevel modeling, to analyze only a limited number of variables based on the theoretical background and researcher’s interest. They do not extensively examine the variables that affect sense of community. Additionally, the main disadvantage of conventional statistical techniques is that as the number of input variables and the number of possible interactions among variables increases, models become more complex and the standard errors of regression coefficients increase (Bzdok et al., 2018).

Machine learning approaches, however, are useful because they facilitate analysis even amid complex, nonlinear interactions and provide opportunities to discover predictors that might not otherwise be identified (Bzdok et al., 2018; Yi and Na, 2019). Hence, to overcome the problems associated with traditional statistical methods and to reveal the important variables that predict high school students’ sense of community, this study adopts random forests (Breiman, 2001), a machine learning technique.

On the other hand, random forests, so-called black boxes that emphasize predictability, cannot provide a simple description of the relationship or direction between predictors and dependent variables due to complicated interactions among the input variables (Buhrmester et al., 2021). Therefore, we have also applied the SHapley Additive exPlanations (SHAP) (Lundberg et al., 2018), i.e., eXplainable Artificial Intelligence (XAI), to examine and interpret the tendencies between the important variables derived through random forests and sense of community.

The following research objectives guided the present study: (a) to explore the key predictors of sense of community among Korean high school students through random forests and (b) to identify the relationships between high school students’ sense of community and these key predictors. For this purpose, the research questions to be confirmed in this study are as follows:

Research Question 1. What are the key predictors of high school students’ sense of community according to random forests?

Research question 2. How is the relationship between the main explanatory variable of high school students’ sense of community derived through random forests and their sense of community?

## 2 Literature review

### 2.1 The concept of sense of community

According to Sarason (1974, p. 1), who first conceptualized the term, sense of community is “the sense that one was the part of a readily available, mutually supportive network of relationships upon which one could depend and as a result of which one did not experience sustained feelings of loneliness.” McMillan and Chavis (1986, p. 9) later proposed a comprehensive theory of sense of community: “the feeling of belonging or of sharing a sense of personal relatedness, a sense of mattering to one another and to the group, the feeling that members” needs will be met by the resources received through their membership in the group, and the belief that members have shared and will share history and similar experiences’.

### 2.2 Factors predicting sense of community

Studies investigating the variables that influence sense of community have reported a range of factors, which can be categorized as student-, family-, or school-related factors.

#### 2.2.1 Student-related factors

Research examining “gender” differences in sense of community has yielded mixed results. Some studies have reported greater levels of sense of community among male students (Kim and Kim, 2015; Leiva et al., 2021), others have shown greater levels of sense of community among female students (Kissinger et al., 2009; Park, 2019), and others have indicated no significant gender differences (Wilkinson, 2008; Park et al., 2015). The self-related variables of “self-efficacy” and “self-esteem” are positively associated with students’ sense of community (Park, 2021; Chon and Kim, 2022). “Career maturity” (Jung et al., 2018), “multicultural acceptance” (Choi and Lee, 2021), and “life satisfaction” (Cantarero et al., 2007) have also been defined as predictors of students’ sense of community.

Students’ “academic achievement” (Wighting et al., 2015; Yi et al., 2017) is also positively associated with their sense of community. Additionally, the more satisfied students are with their school life and the better they have adjusted to school, the greater their sense of community is (Albanesi et al., 2007; Heo, 2020). On the other hand, higher “internet addiction” (Min, 2017) and “mobile phone dependence” (Kim and Lee, 2020) are negatively associated with a sense of community.

#### 2.2.2 Family-related factors

In terms of family demographic characteristics, higher levels of household income and parental education have positive effects on children’s sense of community (Zaff et al., 2008; Koo and Yoo, 2021).

The impact of parents–child relationships is positively related to children’s sense of community (Yi et al., 2017; Kim and Kim, 2018). Family support also influences children’s sense of community. Vieno et al. (2007) noted that “parental social support” plays a significant role in maintaining interpersonal relationships outside the home, even though the main elements of school life are mostly determined by teachers and peers. In addition, Kwak (2017) found that students who perceive high “parental educational support” tend to have a greater sense of community, while Liu et al. (2022) revealed that “parents” emotional support’ is positively related to students’ sense of community. Furthermore, Kuperminc et al. (2008) reported that students’ perceived “parental school involvement” positively contributes to their sense of community. Moreover, positive “parental rearing behavior,” where parents nurture their children with attention and affection, is an important factor in improving children’s sense of community (Park, 2019). Students who report more “parental monitoring,” which refers to the degree to which parents are interested in and observe their children’s leisure time and friendships, also tend to have a greater sense of community; students who perceive more “parental control,” which means the degree to which parents seek to control every aspect of their children’s lives, tend to have a lower sense of community (Vieno et al., 2005).

#### 2.2.3 School-related factors

Given that their school is where students spend the most time, students’ teachers and peers at school, as well as the variables related to school climate, influence their sense of community.

Several studies have indicated that greater “teacher academic pressure” and “teacher enthusiasm” are positively associated with students’ sense of community (Koo and Yoo, 2021). Specifically, Dewaele and Li (2021) revealed that the more students perceive their teachers’ enthusiasm, the greater their social-behavioral engagement. Additionally, a positive “relationship between teacher and student” improves students’ sense of community (Jung, 2020).

Furthermore, greater “social support from peers” is positively related to a greater sense of community (Park et al., 2015; Hombrados-Mendieta et al., 2019). Vieno et al. (2005) showed that “school-level mean socioeconomic status (SES),” “father’s education level and occupational status” and “democratic school climates,” e.g., freedom of expression, perceived rule fairness and equal student treatment are important factors driving students’ sense of community. However, the structural characteristics of schools, such as “school size,” “school sector,” and “school physical facilities,” are not statistically significant. Moreover, a “schools” pro-human rights culture’ increases students’ civil consciousness (Kim and Kim, 2015), while students’ level of school violence is negatively related to their sense of community (Yoon et al., 2018). The quantity of experiential activity time and the degree of satisfaction with adolescent extracurricular activities are also positively related to a sense of community (Shin and Chun, 2017; Cho and Han, 2019).

## 3 Methods

### 3.1 Participants

The current study used data from the 2013 Korean Education Longitudinal Study (KELS2013) to explore the factors that affect high school students’ sense of community. The KELS2013, conducted by the Korean Educational Development Institute, is a panel dataset that contains actual data on students’ educational experiences and outcomes and provides fundamental information for the establishment and evaluation of educational policies. Specifically, the KELS2013 data were collected from 7,324 5th graders attending 242 elementary schools across the country who were chosen by stratified cluster random sampling in 2013. In this study, only data from the 7th wave (11th graders, second year of high school) were used. Students who did not respond to the item measuring sense of community were excluded from the analysis. Thus, the final sample consisted of 6,077 students, and their demographic characteristics were as follows: 3,012 boys (49.6%) and 3,065 girls (50.4%). Among the schools, 16.7% were located in metropolitan areas, 24.5% were located in major cities, 41.6% were located in small to medium-sized cities, and 17.2% were located in rural areas. Additionally, 55.3% of the participants were enrolled in public schools, and 44.7% were enrolled in private schools, 59.8% were enrolled in coeducational schools, 19.7% were enrolled in boys’ schools, and 20.5% were enrolled in girls’ schools.

### 3.2 Variables

The dependent variable was sense of community based on 12 items, e.g., “When I grow up, I will participate in elections and vote;” “I care about others before me;” and “I help my friends who are behind in school,” measured using a self-report scale. These items were rated on a 5-point Likert scale ranging from 1 (strongly disagree) to 5 (strongly agree), and the mean of the 12 questions on sense of community was used in the analysis, with higher scores representing a greater sense of community. Using the Cronbach’s alpha coefficient formula (Cronbach, 1951), the Cronbach’s alpha for sense of community was calculated based on a value of 0.878. This result exceeded the acceptable threshold of 0.7 (Nunnally, 1978), indicating a satisfactory level of reliability.

A total of 159 predictive variables were included in the present study (80 student-, 48 family-, and 31 school-related variables that may directly or indirectly affect sense of community). The specific list of variables is shown in Table 1, and the data preprocessing procedure used for the predictors is described below.

**Table 1:** Predictive variables used in the random forests model.

Category		Variable name	Scale
Student-related	Individual item	Gender	0 = Boy, 1 = Girl
		Class concentration (Korean, math, English)	1 (10 Min or less) ~ 5 (41 Min or more)
		Class comprehension (Korean, math, English)	1 (20% or less) ~ 5 (81% or more)
		Participation in volunteer activities, preparatory education (Math, English) Experience in part-time work School transfer status	0 = No, 1 = Yes
		Reading volume	0 (Unread) ~ 10 (Volume 10 or more)
		Study time	0 (None) ~ 6 (6 h or more)
		Exercise time	0 (None) ~ 9 (9 h or more)
		Leisure activities (participating in after-school programs, doing school homework, doing tutoring homework, attending tutoring lesson, attending online lesson, reading books, watching television, spending time with friends, helping with household chores)	0 (None) ~ 4 (3 h or more)
		Computer/smart media usage time (study/homework, non study information search and resource utilization, text/chat/message/email/call, game/entertainment, club activities/online cafes/community engagement)	0 (None) ~ 5 (3 h or more)
		Educational aspirations	1 (Middle school) ~ 5 (Ph.D.)
		College enrollment plans	0 = Outside the capital (Seoul, Korea), 1 = The capital (Seoul, Korea)
		Expected monthly income after employment	1 (Less than 1 million KRW) ~ 10 (5 Million won or KRW)
	Average	Self-concept (social, family, physical, academic) Creativity Self-management Observance of rules Multicultural acceptance (perceptions of foreigners, relationships with multicultural neighborhood and friends) Mental health Class attitude Relationship with teachers Peer attachment Exam stress Academic stress Positive recognition for volunteering Reading preference Smartphone dependence Career maturity (self-understanding, job planning, attitude toward work)	1 (Strongly disagree) ~ 5 (Strongly agree)
		Frequency of cultural activities	0 (None) ~ 5 (Once or twice a week)
		Intrinsic motivation (Korean, math, English) Self-determined motivation (external motivation, introjected motivation, Identified motivation, intrinsic motivation, amotivation) Academic achievement goal orientation (mastery approach, mastery avoidance, Performance approach, performance avoidance) Self-efficacy (Korean, math, English) Cognitive self-regulated learning strategies (rehearsal, elaboration, clustering, meta-cognition) Behavioral regulation strategy (effort regulation, time management, space management, requesting help and utilizing resources) Preference for cooperative learning Preference for competitive learning, meta-cognition	1 (Strongly disagree) ~ 4 (Strongly agree)
Family-related	Individual item	Coresiding family (father, mother, brothers, sisters, grandparents, relatives) Engagement in private tutoring Experience of activities for career decisions (counseling with the homeroom teacher, counseling with the private tutors, counseling with relatives/neighbors/etc., trying career-related assessments, visiting to advanced schools, gathering information through mass media (TV, newspapers, etc.), gathering information through online communities, purchasing career-related books and web search, participating in career explanation sessions) Experience studying abroad Experience attending school located abroad Parental involvement in school organization activities (joining and participating in the school’s parent association, volunteering at the school, attending parent conferences, observing open classes, attending parent education sessions)	0 = No, 1 = Yes
		Academic expectations for the child	1 (Middle school) ~ 5 (Ph.D.)
		Aspirations for the child’s college enrollment	0 = Outside the capital (Seoul, Korea), 1 = The capital (Seoul, Korea)
		School satisfaction (overall satisfaction)	1 (Strongly disagree) ~ 5 (Strongly agree)
		Child’s height	cm
		Child’s weight	kg
		Monthly household income Monthly educational expenses (all children, surveyed child)	KRW
		Use of Korea Educational Broadcasting System education programs Use of online home-learning program Activities to understand the child’s school life (contacting teachers through phone calls/messages/emails, counseling with school teachers, posting opinions or questions on the school website bulletin board, checking the child’s class bulletin board, viewing the child’s school records)	1 (None) ~ 5 (Everyday)
	Average	Child-perceived parental interaction Child-perceived parental support (academic achievement, emotional well-being) Child-perceived feelings of alienation from parents Parent-perceived parental support (academic achievement, emotional well-being) School satisfaction (school safety, school activities) Relationships with those who share information about the child	1 (Strongly disagree) ~ 5 (Strongly agree)
		Activities with the child	0 (None) ~ 3 (5 Times or more)
School-related	Individual item	School sector 1	0 = Public, 1 = Private
		School sector 2	0 = Coeducation, 1 = Single gender
		Degree of reflecting the student survey results	1 (Strongly disagree) ~ 5 (Strongly agree)
		Experience as a class officer this year Participation in career and occupational programs (counseling, classes, seminars, visits to colleges, field trips, on-site job experiences, virtual job experiences) After-school programs (Korean, math, English, arts/physical) Participation in club activities (academic learning, science research, art/craft, music, sports, newspaper/broadcasting, leisure/game, youth organization, volunteering)	0 = No, 1 = Yes
	Average	Classroom atmosphere Teaching method (individualized, interactive) Teacher characteristics (achievement pressure, teacher enthusiasm) School violence (degree of violence within the school, personal experience of being a victim of violence)	1 (Strongly disagree) ~ 5 (Strongly agree)

### 3.3 Data preprocessing

For the feature analysis, we used the average of each subfactor or the individual items, and the categorical items from the KELS2013 questionnaires were dummy-coded. In addition, for certain items, observations with multiple responses, no response, or an “I do not know” response were considered missing. Additionally, for ease of interpretation, some items were reverse-coded (e.g., “I often choose to watch TV or play before I do my homework” in self-management; “More foreigners will lead to more crimes” in multicultural acceptance). Among the parental variables, monthly household income and monthly educational expenses were utilized in the analysis after taking the natural logarithm. Students who did not respond to the questions requesting information about their school sector and coeducation were excluded from the analysis (*n* = 70). Next, variables with more than 20% missing data were removed from the analysis; for other missing data, single imputation was performed using the “mice” package (Ver, 3.14.0) in R 4.2.2 (van Buuren and Groothuis-Oudshoorn, 2011).

### 3.4 Data analysis

In this study, we employed random forests, a machine learning technique, to explore the important variables that predict high school students’ sense of community. Random forests are ensemble methods based on the decision tree method and bagging (bootstrap aggregating). In random forests, students were randomly sampled with replacement to generate numerous bootstrap samples, and the final prediction was determined by aggregating the results of all the singular decision trees obtained from each bootstrap sample (Breiman, 2001).

The steps in applying random forests were as follows. First, all the data were randomly divided into two sets: a training set (70% of the sample) and a test set (30% of the sample). The random forests model was built with the training data. Second, when generating the individual decision trees, we set the number of variables to be selected at each node to 53 (number of predictors/3 for regression trees) in accordance with Breiman’s (2001) recommendation. Third, to perform hyperparameter tuning for the random forests model, we applied a grid search via scikit-learn’s GridSearchCV (Ver, 1.0.2 for Python 3.9). The number of trees was chosen from 100, 300, 500, 700, 1,000 and 1,500. Based on 10-fold cross-validation, the optimal hyperparameter that produced the best performance metric (highest R-Squared) was selected (Snider et al., 2021). Fourth, to evaluate the performance of the random forests model, we calculated the root mean squared error (RMSE) and R-squared (*R*^2^) on the test set, which are typical evaluation metrics for regression models.

After the random forests, the SHAP value was derived to determine the importance of each feature, which indicates its relative contribution to the model’s prediction. SHAP is based on coalitional game theory, which can help explain the output of complex machine learning models (Lundberg and Lee, 2017; Lundberg et al., 2018). We derived the top 10 key variables selected by the mean absolute Shapley values, which contributed most significantly to predicting the sense of community among high school students. To interpret the relationships between the key predictors and the dependent variable, sense of community, we constructed a SHAP summary plot and SHAP dependence plots. The random forests were conducted using the RandomForestRegressor class from the scikit-learn libraries in Python 3.9, and the SHAP algorithm was applied using the TreeExplainer class from the SHAP library.

## 4 Results

### 4.1 Model tuning

After performing a grid search using 10-fold cross-validation, it was revealed that the highest cross-validation score occurred when the number of decision trees was set to 700, which was used to generate the random forests model. The final model, incorporating the optimal hyperparameters, exhibited an RMSE of 0.333 and an *R*^2^ of 0.680.

### 4.2 Feature importance

**Figure 1:**
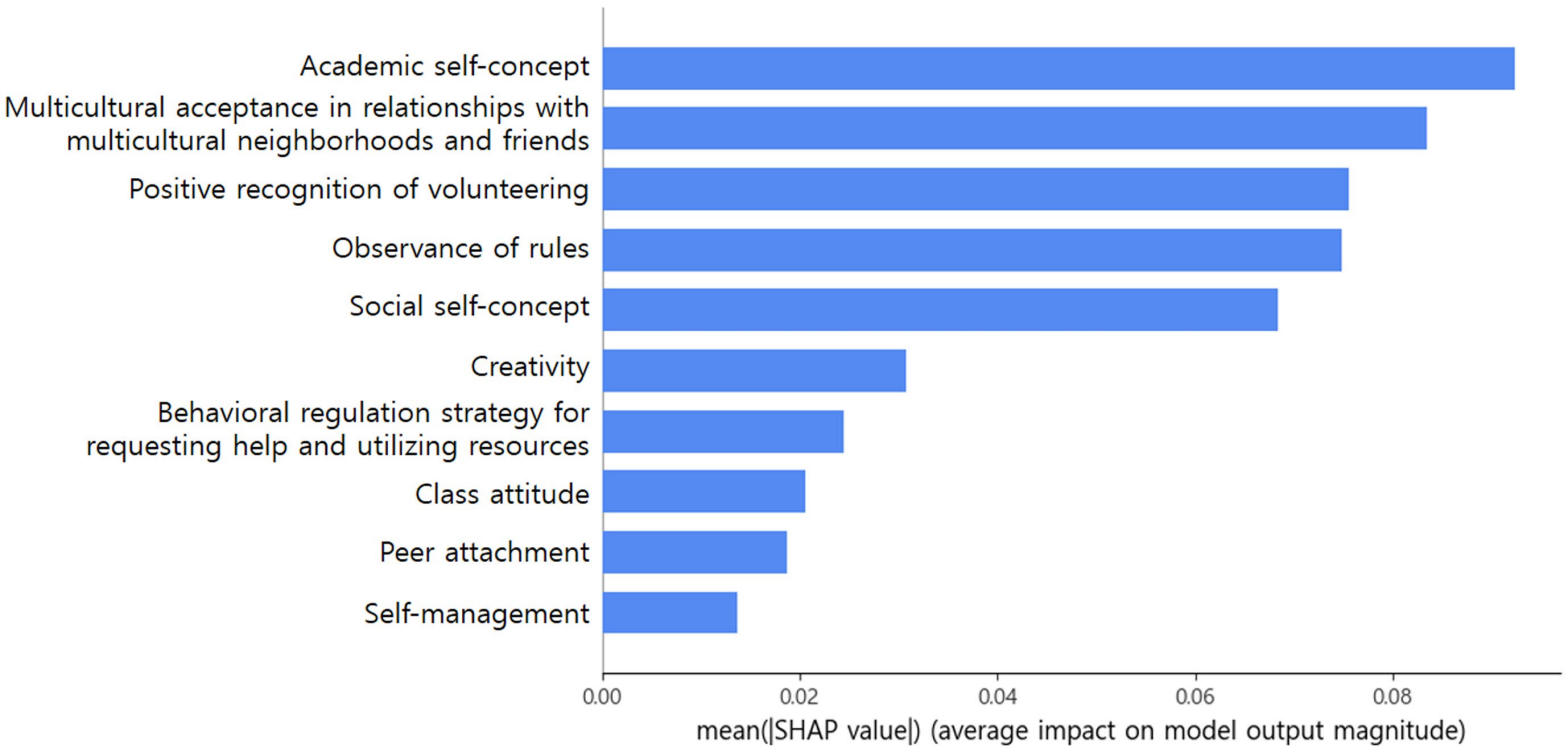
Average absolute SHAP values for the top 10 features ordered by feature importance.

After fitting the random forests, the absolute average of the SHAP values was calculated to assess the relative importance of the features in predicting the sense of community. Figure 1 shows the top-10 features with the highest feature importance in descending order, and the horizontal length of each bar indicates the magnitude of the average contribution to the model (Taye et al., 2023). As shown in Figure 1, “academic self-concept” was derived as the variable most relevant to high school students’ sense of community. Next, “multicultural acceptance in relationships with multicultural neighborhoods and friends,” “positive recognition of volunteering,” “observance of rules,” “social self-concept,” “creativity,” “behavioral regulation strategy for requesting help and utilizing resources,” “class attitude,” “peer attachment” and “self-management” were ranked as the top 10 variables according to feature importance (average absolute Shapley value), suggesting that these features contribute the most significantly to predicting individual sense of community.

### 4.3 Feature interpretation

**Figure 2:**
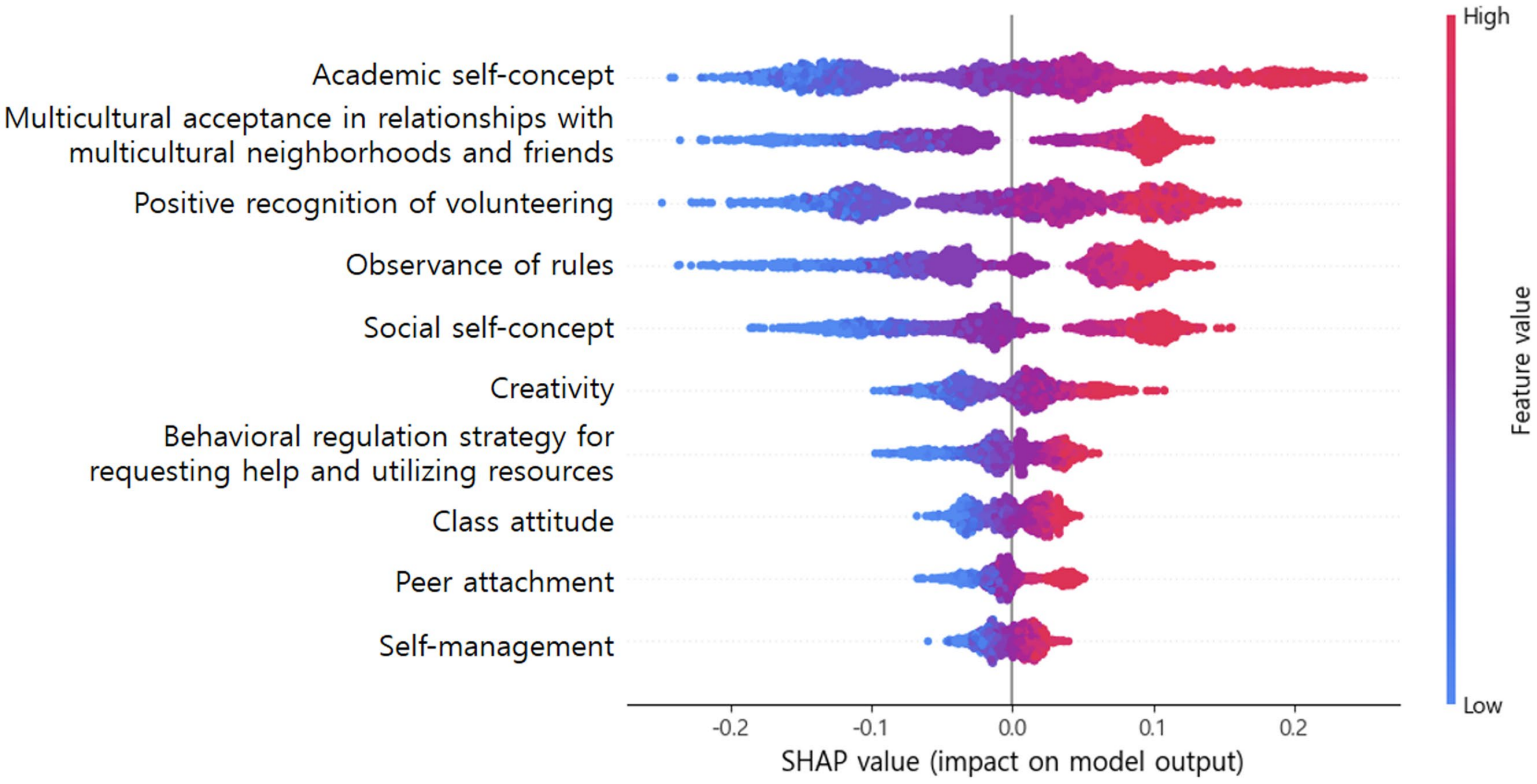
SHAP summary plot with the top 10 features ordered by feature importance.

To understand the directionality of the influence of the top 10 features on high school students’ sense of community, we constructed the SHAP summary plot shown in Figure 2. The SHAP summary plot combines the feature importance and direction of the impact of each feature on the sense of community (Molnar, 2022). The y-axis displays the top 10 predictors of sense of community, ranked in order of average influence on the prediction, and the x-axis describes the Shapley value related to each feature. Individual-negative Shapley values extending to the left are interpreted as decreased levels of sense of community; positive Shapley values extending to the right are interpreted as increased levels of sense of community (Taye et al., 2023). Each dot on the SHAP summary plot corresponds to the Shapley value of a feature of an individual with different colors; red dots represent high feature values, and blue dots represent low feature values. For instance, Figure 2 shows that “academic self-concept” is the most important feature for predicting a sense of community, and has a positive impact on predicting a sense of community because the red dots representing high values of the variable are located to the right of the y-axis with a SHAP value of 0. The other nine variables also show a gradual transition from blue to red along the vertical axis, indicating that all the other predictors have a positive impact on the sense of community, since the level of SHAP increases as the values of all the variables increase.

**Figure 3:**
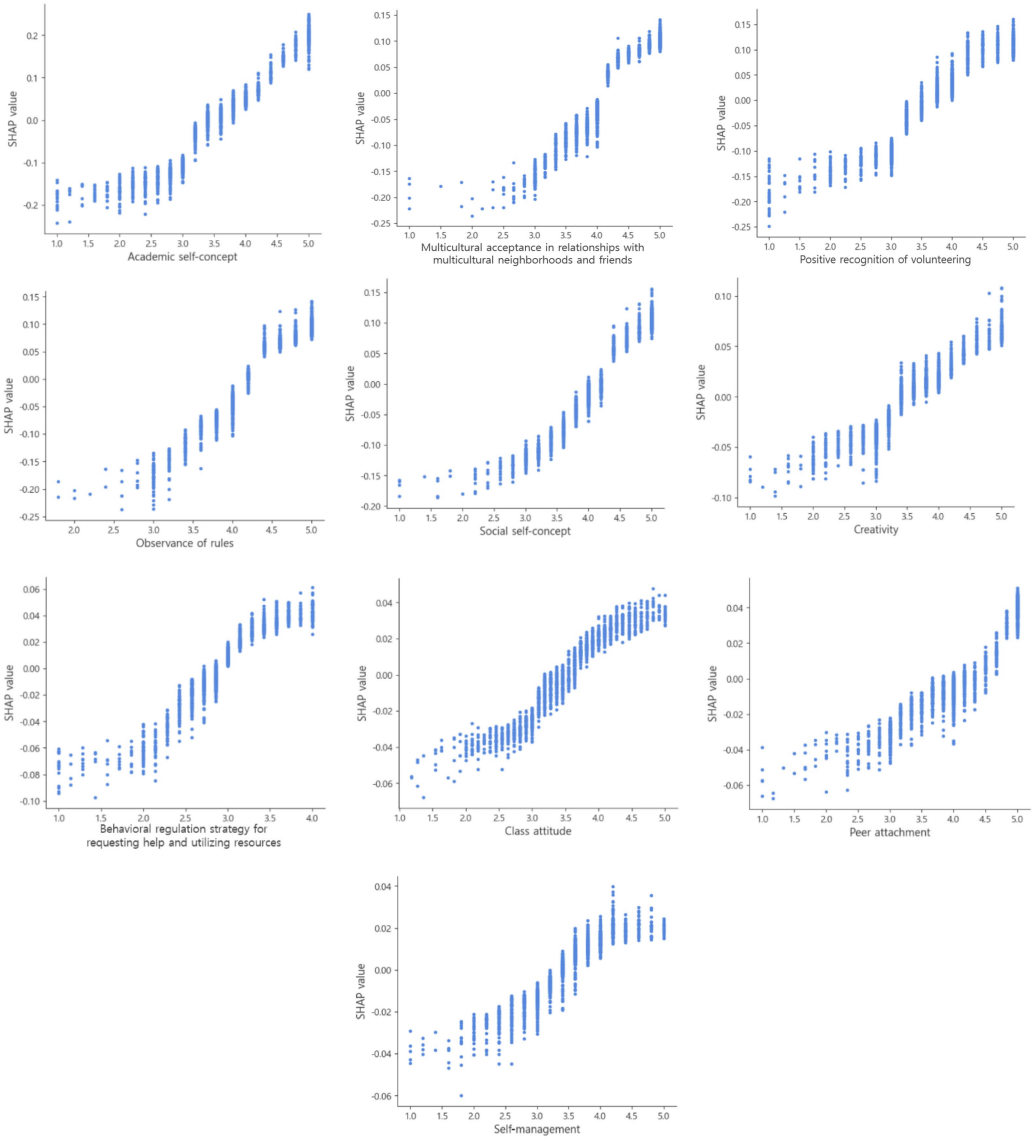
SHAP dependence plot illustrating the distribution of Shapley values.

In Figure 3, the exact relationships between the top 10 predictors and sense of community are visualized through the SHAP dependence plot. The SHAP dependence plot is a scatter plot of predictors and Shapley values, where the x-axis indicates the raw values of each predictor and the Shapley value of the same predictor is on the y-axis (Lundberg et al., 2020; Zhang et al., 2022). Figure 3 shows that as the raw value of “academic self-concept” increases from 1 to 5, the SHAP value also shifts from negative to positive. This can be interpreted as indicating a positive relationship between the two variables, with higher academic self-concept tending to be associated with a greater sense of community. The other nine predictors also showed that as the raw value increased, the SHAP value tended to increase, suggesting positive relationships between the predictors and sense of community.

## 5 Discussion

The aim of this study was to reveal the top 10 predictor variables with the highest impact on high school students’ sense of community based on 159 variables in the KELS2013 study and by using random forests, a machine learning technique, and to visualize and interpret the relationship between these key predictors and sense of community by applying the SHAP algorithm. This section therefore elaborates on the novel findings of the current study and compares them with relevant results in the literature.

First, student-related factors such as “self-concept,” “self-management,” “multicultural acceptance,” “positive recognition for volunteering” and “creativity” are the key variables predicting high school students’ sense of community. Specifically, self-related variables such as “academic self-concept,” “social self-concept” and “self-management” are the top variables with a positive relationship with sense of community. These results are supported in the literature (Park, 2021;Chon and Kim, 2022). Therefore, it is important for schools to recognize the importance of intrapersonal variables in improving a sense of community and encouraging students to develop a healthy self-concept and perceive themselves positively.

Second, “multicultural acceptance in relationships with multicultural neighborhoods and friends” is an important variable for sense of community, and “multicultural acceptance” is also positively related to the latter. Considering that multicultural acceptance and sense of community are concepts that emphasize living as a community while recognizing the diversity of individuals (Baek and Chung, 2017), these two variables are closely related. Thus, it is necessary to expand the scope of education from the individual to the social level and to provide a school environment where students can interact with diverse people to improve their sense of community. On the other hand, previous studies have provided mixed results concerning the causal relationship and direction between “multicultural acceptance” and sense of community. Some studies indicate that “multicultural acceptance” affects sense of community (Park, 2021); others suggest that sense of community influences “multicultural acceptance” (Choi, 2019). Consequently, in-depth and further research is needed to explore and clarify the relationships between these two variables.

Third, “positive recognition of volunteering” and “creativity” are key variables with a positive relationship to sense of community; they are remarkable in that they are novel variables. Rhoads (1998) suggested that community service involves interaction with a variety of others and that in this process, community service serves as a vehicle for creating communal ties. In addition, since the “positive recognition of volunteering” item in this study measures attitudes toward voluntary and active engagement in volunteer activities, “positive recognition of volunteering,” which intrinsically motivates them to continue to volunteer, leads to an increase in students’ sense of community.

Moreover, some studies have reported that openness and extraversion are strongly related to creativity (Kaufman et al., 2016; Lebedeva et al., 2018; Miroshnik et al., 2022). Accordingly, there is likely a relationship between “creativity” and a sense of community that emphasizes a community’s willingness to embrace diversity (Kim et al., 2020). In other words, students with higher levels of “creativity” are more likely to possess a diverse range of ideas and open-minded perspectives, which may foster their ability to respect and embrace differing opinions among others.

Fourth, “behavioral regulation strategy for requesting help and utilizing resources” and “peer attachment” are key variables related to sense of community and are positively related to it. The “behavioral regulation strategy for help and resource utilization” in this study entails seeking help from teachers and friends when in trouble (e.g., “If there’s something I’m not sure about, I ask an acquaintance”), and “peer attachment” represents forming positive relationships with friends by, for example, trusting them and confiding in them (e.g., “I can tell my friends what’s on my mind”). Given that the variables related to relationships with others are key predictors of sense of community, these variables may be important for living with community members in a communal society. Additionally, these findings are consistent with the literature (Kang and Jang, 2013; Park et al., 2015; Kim and Kim, 2018; Hombrados-Mendieta et al., 2019), which has demonstrated that positive interpersonal relationships with teachers or peers are positively associated with students’ sense of community.

Fifth, “observance of rules” and “class attitude” are novel key predictors; they are positively related to sense of community. In this study, “observance of rules” refers to keeping promises to others and following class rules, and “class attitude” entails following academic etiquette, such as paying attention in class and doing well on assignments. Therefore, not only maintaining good relationships as a member of a school community but also maintaining common rules and regulations are important for the formation of a sense of community.

## 6 Conclusion and future directions

While studies on variables that influence sense of community have examined only limited variables, based on their theoretical background or literature review, with conventional statistical techniques, the present study meaningfully identified numerous variables that have not been considered in the literature, as the key predictors of sense of community. Hence, this study is valuable because it reveals which variables should be considered important in fostering a sense of community among high school students by using random forests, a machine learning technique. Additionally, the present study has contributed to the literature through its novel application of SHAP to overcome the disadvantage of random forests in terms of the difficulty of interpreting their results, illustrating the relationship between sense of community and its key predictors by visualizing them.

This study has several limitations. First, this study is limited by its exclusive utilization of data collected from Korean students surveyed within Korea. Subsequent research is therefore expected to provide richer insights into sense of community by conducting cross-national comparative studies that utilize large-scale international survey data to explore the cultural context characteristics related to the formation of sense of community. Second, this study used cross-sectional data, which limits the exploration of developmental factors explaining changes in sense of community, and caution should be exercised when interpreting the causal relationships among variables. Therefore, future studies could explore the variables related to changes in the sense of community from a longitudinal perspective.

## Data availability statement

Publicly available datasets were analyzed in this study. This data can be found at: https://www.kedi.re.kr/khome/main/research/requestResearchData.do.

## Ethics statement

The studies involving humans were approved by Institutional Review Board in Korean Educational Development Institute (IRB number: 2019-16-05-N). The studies were conducted in accordance with the local legislation and institutional requirements. Written informed consent for participation was not required from the participants or the participants’ legal guardians/next of kin in accordance with the national legislation and institutional requirements.

## Author contributions

EJ: Writing – original draft, review & editing. HC: Writing – original draft, review & editing.

## References

[ref1] Albanesi C. Cicognani E. Zani B. (2007). Sense of community, civic engagement and social well-being in Italian adolescents. J. Community Appl. Soc. Psychol. 17, 387–406. doi: 10.1002/casp.903

[ref2] Baek S. Chung H. (2017). The developmental trajectories of multicultural acceptance and the changing community spirit as determined by means of growth mixture modeling. Stud. Korean Youth 28, 151–182. doi: 10.14816/sky.2017.28.1.151

[ref3] Breiman L. (2001). Random forests. Mach. Learn. 45, 5–32. doi: 10.1023/A:1010950718922

[ref4] Buhrmester V. Munch D. Arens M. (2021). Analysis of explainers of black box deep neural networks for computer vision: a survey. Mach. Learn. Knowl. Extr. 3, 966–989. doi: 10.3390/make3040048

[ref5] Bzdok D. Altman N. Krzywinski M. (2018). Statistics versus machine learning. Nat. Methods 15, 233–234. doi: 10.1038/nmeth.4642, PMID: 30100822 PMC6082636

[ref6] Cantarero R. Potter J. J. Leach C. K. (2007). Perceptions of quality of life, sense of community, and life satisfaction among elderly residents in Schuyler and Crete, Nebraska. Archit. Program Fac. Sch. Creat. Act. 4, 35–40.

[ref7] Cho Y. S. Han S. Y. (2019). Relationship between satisfaction with adolescent extracurricular activities and social withdrawal: the mediating effect of self-esteem and sense of community. Fam. Environ. Res. 57, 243–255. doi: 10.6115/fer.2019.017

[ref9002] Choi J. (2019). The relationship of multicultural acceptance, community closeness, self-awareness, and interpersonal relationship: A structural equation modeling approach. Int. J. Innov. Tech. Explor. Eng. 8, 5–11.

[ref8] Choi E. J. Lee K. (2021). Analysis of longitudinal relationship among elementary and middle school students’ multicultural acceptance, self-concept, and community consciousness using the latent growth model. Int. Electron. J. Elem. Educ. 13, 565–575. doi: 10.26822/iejee.2021.212

[ref9] Chon M. Kim M. (2022). Factors affecting the sense of community for women in community participatory health services. J. ReAtt. Ther. Dev. Divers. 5, 224–228.

[ref10] Cronbach L. J. (1951). Coefficient alpha and the internal structure of tests. Psychometrika 16, 297–334. doi: 10.1007/BF02310555

[ref11] Dewaele J. Li C. (2021). Teacher enthusiasm and students’ social-behavioral learning engagement: the mediating role of student enjoyment and boredom in Chinese EFL classes. Lang. Teach. Res. 25, 922–945. doi: 10.1177/13621688211014538

[ref12] Erikson E. (1968). Youth: Identity and Crisis. New York: W.W. Norton.

[ref13] Heo H. (2020). Relationship between school adjustment and sense of community in adolescents using autoregressive cross-lagged model. J. Fish. Mar. Sci. 32, 866–877. doi: 10.13000/JFMSE.2020.6.32.3.866

[ref14] Hombrados-Mendieta I. Millan-Franco M. Gomez-Jacinto L. Gonzalez-Castro F. Martos-Mendez M. J. Garcia-Cid A. (2019). Positive influences of social support on sense of community, life satisfaction and the health of immigrants in Spain. Front. Psychol. 10:2555. doi: 10.3389/fpsyg.2019.02555, PMID: 31803103 PMC6872520

[ref15] Jung M. S. (2020). Effects of child abuse and neglect on community sense of adolescent - focusing on mediated effects of classmate and teacher relations. J. Korea Cont. Assoc. 20, 106–115. doi: 10.5392/JKCA.2020.20.03.106

[ref16] Jung J. E. Kim K. S. Kwak H. (2018). The longitudinal relationship among relationship formation, career maturity, sense of community of early adolescents. Korean J. Elem. Educ. 29, 35–58. doi: 10.20972/kjee.29.1.201803.35

[ref9005] Kang G. Y. Jang Y. M. (2013). A study on sense of community of adolescents. J. futur. Orient. Youth. Soc. 10, 97–123

[ref17] Kaufman S. B. Quilty L. C. Grazioplene R. G. Hirsh J. B. Gray J. R. Peterson J. B.. (2016). Openness to experience and intellect differentially predict creative achievement in the arts and sciences. J. Pers. 84, 248–258. doi: 10.1111/jopy.12156, PMID: 25487993 PMC4459939

[ref18] Kim H. Kim S. (2015). A study on the effects of pro-human rights culture in schools on youths’ civil consciousness. Theory Res. Citizsh. Educ. 47, 29–52. doi: 10.35557/trce.47.2.201506.002

[ref19] Kim D. Kim T. (2018). The effects of social relationships in proximal contexts on adolescents’ sense of community and multicultural acceptance. Theory Res. Citizsh. Educ. 50, 23–47. doi: 10.35557/trce.50.3.201809.002

[ref20] Kim J. Lee H. (2020). Analysis of the mediating effects of social withdrawal in the relationship between mobile phone dependency and the sense of community among adolescents. J. Digit. Converg. 18, 273–279. doi: 10.14400/JDC.2020.18.4.273

[ref21] Kim M. Yu Y. Hwang E. (2020). A study on influential factors on adolescents’ sense of community using data mining: a comparison between middle and high school students. Stud. Korean Youth 31, 205–233. doi: 10.14816/sky.2020.31.2.205

[ref22] Kissinger J. Campbell R. C. Lombrozo A. Wilson D. (2009). “The role of gender in belonging and sense of community,” in 2009 39th IEEE Frontiers in Education Conference. San Antonio: Institute of Electrical and Electronics Engineers (IEEE), 1–6.

[ref23] Koo M. Yoo J. E. (2021). Piecewise growth modeling of adolescents’ sense of community. Korean J. Educ. Res. 59, 363–388. doi: 10.30916/KERA.59.2.361

[ref24] Kuperminc G. P. Darnell A. J. Alvarez-Jimenez A. (2008). Parent involvement in the academic adjustment of Latino middle and high school youth: teacher expectations and school belonging as mediators. J. Adolesc. 31, 469–483. doi: 10.1016/j.adolescence.2007.09.003, PMID: 17953983

[ref25] Kwak S. R. (2017). An analysis of the determinants of creativity and a sense of community from childhood to adolescence in the context of academic achievement. J. Korean Educ. 44, 5–28. doi: 10.22804/jke.2017.44.4.001

[ref26] Lebedeva N. Schwartz S. Plucker J. Van de Vijver F. (2018). Domains of everyday creativity and personal values. Front. Psychol. 9:2681. doi: 10.3389/fpsyg.2018.02681, PMID: 30692950 PMC6339925

[ref27] Lee S. C. (2006). Research on consciousness of youth volunteering based on holistic education. J. Holist. Educ. 10, 69–87.

[ref9001] Lee M. Cho O. (2023). The effects of civic consciousness and sense of community on happiness in adolescent: Mediating effects of career decision. J. Ind. Converg. 21, 97–107. doi: 10.22678/JIC.2023.21.5.097

[ref28] Leiva L. Mendoza A. Torres-Cortes B. Antivilo-Bruna A. (2021). Relationship between sense of community, well-being, mental health and gender in schooled adolescents. Psicoperspectivas. 20, 41–54. doi: 10.5027/psicoperspectivas-vol20-issue2-fulltext-2205

[ref29] Liu Y. B. Hou X. Y. Chen B. B. (2022). Links between Chinese vocational school students’ perception of parents’ emotional support and school cooperation climate and their academic performance: the mediating role of school belonging. Front. Psychol. 13:952001. doi: 10.3389/fpsyg.2022.952001, PMID: 35967675 PMC9374126

[ref30] Lundberg S. M. Erion G. Chen H. DeGrave A. Prutkin J. M. Nair B.. (2020). From local explanations to global understanding with explainable AI for trees. Nat. Mach. Intell. 2, 56–67. doi: 10.1038/s42256-019-0138-9, PMID: 32607472 PMC7326367

[ref31] Lundberg S. M. Erion G. Lee K. (2018). Consistent individualized feature attribution for tree ensembles. arXiv, 1–9. doi: 10.48550/arXiv.1802.03888

[ref32] Lundberg S. M. Lee S. I. (2017). A unified approach to interpreting model predictions. arXiv, 1–10. doi: 10.48550/arXiv.1705.07874

[ref33] McMillan D. W. Chavis D. M. (1986). Sense of community: a definition and theory. J. Community Psychol. 14, 6–23. doi: 10.1002/1520-6629(198601)14:1<6::AID-JCOP2290140103>3.0.CO;2-I

[ref34] Min J. (2017). A study on the internet abuse factors of community sentiment: Foused on social presence and social interaction. J. Korea Entertain. Ind. Assoc. 11, 153–164. doi: 10.21184/jkeia.2017.01.11.1.153

[ref35] Miroshnik K. G. Shcherbakova O. V. Kaufman J. C. (2022). Kaufman domains of creativity scale: relationship to occupation and measurement invariance across gender. Creat. Res. J. 34, 159–177. doi: 10.1080/10400419.2021.1953823

[ref36] Molnar C. (2022). Interpretable Machine Learning: A Guide for Making Black Box Models Explainable. Available at: https://christophm.github.io/interpretable-ml-book/ (Accessed August 13, 2023).

[ref37] Nunnally J. C. (1978). Psychometric Theory (2nd). New York: McGraw-Hill.

[ref38] Park H. (2019). A five-year longitudinal study of the effect of individual, family, and school characteristics on the development of a sense of community among Korean children. J. Educ. Cult. 25, 555–576. doi: 10.24159/joec.2019.25.1.555

[ref9003] Park H. (2021). A longitudinal study investigating the relationships among multicultural receptivity, sense of community, and self-esteem of Korean adolescents. J. Community. Psychol. 50, 2385–2398. doi: 10.1002/jcop.22782 34969149

[ref9004] Park H. Kim J. Son Y. (2015). The analysis of the longitudinal trend of the sense of community in adolescence and its predictors. Asian J. Educ. 16, 105–127. doi: 10.15753/aje.2015.12.16.4.105

[ref40] Ranju L. Manisha D. (2015). Emotional intelligence and social adaptability. Int. J. Sci. Technol. Manag. 4, 801–807.

[ref41] Rhoads R. A. (1998). In the service of citizenship: a study of student involvement in community service. J. High. Educ. 69, 277–297.

[ref42] Sarason S. B. (1974). The Psychological Sense of Community: Prospects for a Community Psychology. London: Jossey-Bass.

[ref43] Shin I. Chun D. (2017). Causality between youth experience activities and sense of community. Soc. Sci. Res. Rev. 33, 121–140. doi: 10.18859/ssrr.2017.11.33.4.121

[ref44] Snider B. McBean E. A. Yawney J. Gadsden S. A. Patel B. (2021). Identification of variable importance for predictions of mortality from COVID-19 using AI models for Ontario, Canada. Front. Public Health 9:675766. doi: 10.3389/fpubh.2021.675766, PMID: 34235131 PMC8255789

[ref45] Taye A. D. Borga L. G. Greiff S. Vögele C. D’Ambrosio C. (2023). A machine learning approach to predict self-protecting behaviors during the early wave of the COVID-19 pandemic. Sci. Rep. 13:6121. doi: 10.1038/s41598-023-33033-1, PMID: 37059871 PMC10103659

[ref46] Terry R. Townley G. Brusilovskiy E. Salzer M. S. (2019). The influence of sense of community on the relationship between community participation and mental health for individuals with serious mental illnesses. J. Community Psychol. 47, 163–175. doi: 10.1002/jcop.22115, PMID: 30506935

[ref47] van Buuren S. Groothuis-Oudshoorn K. (2011). Mice: multivariate imputation by chained equations in R. J. Stat. 45, 1–67. doi: 10.18637/jss.v045.i03

[ref48] Vieno A. Perkins D. D. Smith T. M. Santinello M. (2005). Democratic school climate and sense of community in school: a multilevel analysis. Am. J. Community Psychol. 36, 327–341. doi: 10.1007/s10464-005-8629-8, PMID: 16389503

[ref49] Vieno A. Santinello M. Pastore M. Perkins D. D. (2007). Social support, sense of community inschool, and self-efficacy as resources during early adolescence: an integrative model. Am. J. Community Psychol. 39, 177–190. doi: 10.1007/s10464-007-9095-2, PMID: 17437191

[ref50] Wighting M. J. Nisbet D. Spaulding L. S. (2015). Relationships between sense of community and academic achievement. Int. J. Humanit. Annu. Rev. 7, 63–72. doi: 10.18848/1447-9508/CGP/v07i03/41368

[ref51] Wilkinson D. (2008). Individual and community factors affecting psychological sense of community, attraction, and neighboring in rural communities. Can. Rev. Sociol. 45, 305–329. doi: 10.1111/j.1755-618X.2008.00013.x, PMID: 19579352

[ref52] Yi K. Bang S. M. Lee E. (2017). A structural equation model of the factors affecting adolescents’ sense of community: focusing on mediating effect of academic achievement and academic related stress. Korean J. Sociol. Educ. 27, 105–135. doi: 10.32465/ksocio.2017.27.4.005

[ref53] Yi H. S. Na W. (2019). How are maths-anxious students identified and what are the key predictors of maths anxiety? Insights gained from PISA results for Korean adolescents. Asia Pac. J. Educ. 40, 247–262. doi: 10.1080/02188791.2019.1692782

[ref54] Yoon S. Lee Y. Im S. Kim E. (2018). A factor analysis of students’ sense of community. Asian J. Educ. 19, 353–374. doi: 10.15753/aje.2018.06.19.2.353

[ref55] Yoon Y. Sung J. (2021). Exploring the predictors of community competency according to gender of high school students: using random forest analysis. Korean J. Youth Stud. 28, 383–410. doi: 10.21509/KJYS.2021.04.28.4.383

[ref56] Zaff J. F. Malanchuk O. Eccles J. S. (2008). Predicting positive citizenship from adolescence to young adulthood: the effects of a civic context. Appl. Dev. Sci. 12, 38–53. doi: 10.1080/10888690801910567, PMID: 22837638 PMC3403711

[ref57] Zhang H. Wang Z. Tang Y. Chen X. You D. Wu Y.. (2022). Prediction of acute kidney injury after cardiac surgery: model development using a Chinese electronic health record dataset. J. Transl. Med. 20:166. doi: 10.1186/s12967-022-03351-5, PMID: 35397573 PMC8994277

